# Marker-Independent Monitoring of *in vitro* and *in vivo* Degradation of Supramolecular Polymers Applied in Cardiovascular *in situ* Tissue Engineering

**DOI:** 10.3389/fcvm.2022.885873

**Published:** 2022-05-17

**Authors:** Julia Marzi, Emma C. Munnig Schmidt, Eva M. Brauchle, Tamar B. Wissing, Hannah Bauer, Aurelie Serrero, Serge H. M. Söntjens, Anton W. Bosman, Martijn A. J. Cox, Anthal I. P. M. Smits, Katja Schenke-Layland

**Affiliations:** ^1^Department for Medical Technologies and Regenerative Medicine, Institute of Biomedical Engineering, Eberhard Karls University Tübingen, Tübingen, Germany; ^2^NMI Natural and Medical Sciences Institute at the University of Tübingen, Reutlingen, Germany; ^3^Cluster of Excellence iFIT (EXC 2180) “Image-Guided and Functionally Instructed Tumor Therapies, ” Eberhard Karls University Tübingen, Tübingen, Germany; ^4^Department of Biomedical Engineering, Eindhoven University of Technology, Eindhoven, Netherlands; ^5^Institute for Complex Molecular Systems (ICMS), Eindhoven University of Technology Eindhoven, Netherlands; ^6^Xeltis BV, Eindhoven, Netherlands; ^7^SyMO-Chem BV, Eindhoven, Netherlands; ^8^SupraPolix BV, Eindhoven, Netherlands; ^9^Cardiovascular Research Laboratories, Department of Medicine, David Geffen School of Medicine at University of California, Los Angeles, Los Angeles, CA, United States

**Keywords:** Raman imaging, carotid implantation, tissue-engineered vascular grafts (TEVG), guided tissue engineering, resorption

## Abstract

The equilibrium between scaffold degradation and neotissue formation, is highly essential for *in situ* tissue engineering. Herein, biodegradable grafts function as temporal roadmap to guide regeneration. The ability to monitor and understand the dynamics of degradation and tissue deposition in *in situ* cardiovascular graft materials is therefore of great value to accelerate the implementation of safe and sustainable tissue-engineered vascular grafts (TEVGs) as a substitute for conventional prosthetic grafts. In this study, we investigated the potential of Raman microspectroscopy and Raman imaging to monitor degradation kinetics of supramolecular polymers, which are employed as degradable scaffolds in *in situ* tissue engineering. Raman imaging was applied on *in vitro* degraded polymers, investigating two different polymer materials, subjected to oxidative and enzymatically-induced degradation. Furthermore, the method was transferred to analyze *in vivo* degradation of tissue-engineered carotid grafts after 6 and 12 months in a sheep model. Multivariate data analysis allowed to trace degradation and to compare the data from *in vitro* and *in vivo* degradation, indicating similar molecular observations in spectral signatures between implants and oxidative *in vitro* degradation. *In vivo* degradation appeared to be dominated by oxidative pathways. Furthermore, information on collagen deposition and composition could simultaneously be obtained from the same image scans. Our results demonstrate the sensitivity of Raman microspectroscopy to determine degradation stages and the assigned molecular changes non-destructively, encouraging future exploration of this techniques for time-resolved quality assessment of *in situ* tissue engineering processes.

## Introduction

Current cardiovascular grafts are limited in their ability to replace native tissue. Hemodynamic changes require to adapt continuously, which is difficult to be artificially reproduced (1). As a result, complications often occur due to incompatibility between the native tissue and the grafted material (2). Tissue engineered grafts have been considered as promising cardiovascular replacements, but require time for cell harvesting, conditioning and production of rigid matrix, which is why these are not applicable for off-the-shelf usage (1). *In situ* tissue engineering or endogenous tissue restoration might be the solution, as in this method a degradable biomaterial is implanted in the patient to temporarily function as both, graft and scaffold for endogenous cells, directing regeneration directly at the intended site in the body (3). Biomaterials applied in *in situ* tissue engineering range from polymer scaffolds to *in vitro* generated matrices and decellularized xeno- or allografts (4–6). Over time the biomaterial will degrade, while matrix is produced by the cells that infiltrate and populate the scaffold. These two processes should happen at similar rates to preserve the integrity of the graft. Here, supramolecular materials enable the introduction of different molecular groups and so allow for tailoring of material characteristics, including degradation rates and mechanisms (7). Supramolecular materials can form stacks, as hydrogen bonds are formed between the supramolecular groups, which are non-covalent and therefore dynamic (7). This also enables integration of specific functional groups in a polymer. Under ideal conditions all biomaterial will degrade and a living tissue will remain with the ability to grow and adjust to its environment (8).

Implanted biomaterials degrade due to several processes, namely (1) enzymatic hydrolysis, also called enzymatic degradation when catalyzed by enzymes such as protease and esterase; (2) oxidation caused by reactive oxygen species (ROS) and (3) physical deterioration caused by water absorption (9). The immune system influences both enzymatic and oxidative degradation by way of macrophages and other activated immune cells, which secrete enzymes and ROS when in contact with the biomaterial scaffold (10). On a molecular level, hydrolytic degradation occurs when water molecules substitute ester bonds in a polymer (9). This causes breaking of the polymer chains and results in smaller polymer segments. In oxidative degradation, oxygen-comprising free radicals such as hydroxide or superoxide radicals react with the polymer (9). Especially, methylene protons next to functional groups in the polymer backbone are sensitive to hydrogen extraction, ultimately resulting in chain scission (or possibly cross-linking) of the degraded polymer. Typical molecules generated in oxidative degradation are water, carbon dioxide, and oligomeric chains with hydroxyl chain ends and/or carboxylic acid chain ends. Tissue engineered grafts will be constantly load-bearing during the transition from biomaterial to tissue. Accordingly, an appropriate degradation rate is of utmost importance for the success of the *in situ* procedure (9). Therefore, there is a need for tailoring the degradation kinetics of the used scaffold material. Furthermore, the biocompatibility and potential cytotoxicity of degradation products are an essential safety aspect of biodegradable polymers (11). Thus, unraveling the mechanisms of polymer degradation and potential byproducts on a molecular level and correlating *in vitro* observations to *in vivo* processes is highly relevant for the design of successful implant materials providing long-term functionality as well as biocompatibility and immunocompatibility.

Methods to assess graft degradation *in vitro*, include scanning electron microcopy (SEM), gel permeation chromatography (GPC) or thermal and mechanical analyses to determine morphological changes and polymer integrity and have been thoroughly applied to study polymer degradation (10, 12, 13). These methods can similarly be applied for the analysis of *in vivo* degradation but require extensive sample processing or the removal of the implant material from the surrounding tissue and often do not allow to obtain spatially resolved information. Gaining spatial information on the local material degradation is highly important given that scaffold degradation is subject to strong spatiotemporal heterogeneities, due to differences in local microenvironment such as variations in cell influx or local hemodynamic loads (14, 15).

For more than two decades, material science employs Raman spectroscopy, but in the field of biological tissues this laser-based technique is relatively new (16, 17). Raman microspectroscopy is of great interest for both *in vitro* and *in vivo* applications due to its non-invasive nature and ease of sample preparation in comparison to more conventional methods, as it is label-free. Similar to infrared spectroscopy, which has been applied in a few studies to investigate *in vitro* as well as *in vivo* polymer degradation (18–20), Raman microspectroscopy allows for the non-destructive detection of molecular changes in both, synthetic as well as biological molecules, and in addition enables the simultaneous visualization of their spatial distribution in Raman imaging setups. In comparison to local polymer degradation, extracellular matrix formation and cellular infiltration in *in situ* tissue engineering can be studied at a spatial resolution by histological methods (21, 22), and were also in the focus in previous publications on *in situ* tissue-engineered heart valves and carotid implants, where Raman imaging was implemented as complementary method to routine histochemical and immunofluorescence staining (5, 15).

The aim of this study is to gain a better understanding of *in vivo* degradation pathways of supramolecular polymers which are promising candidates for *in situ* tissue engineering. Raman microspectroscopy and imaging were established in combination with multivariate analysis to assess degradation *in situ* and avoid further sample destruction by preprocessing steps. Molecular fingerprints of oxidative and enzymatic degradation were determined in fast and slow degrading polymer composites and transferred for the analysis of *in vivo* degradation in a carotid implant in a sheep model monitored over a period of 12 months.

## Materials and Methods

### Polymer Scaffolds and Films

Polycaprolactone_2000_-Bisurea (PCL-BU) material (SyMO-Chem, Eindhoven, The Netherlands) and Poly(hexamethylene-) carbonate-Ureido-Pyrimidinone (PC-UPy) material (Xeltis, Eindhoven, The Netherlands) were prepared and electrospun to form tubular scaffolds of 6–23 mm inner diameter. Scaffolds were fabricated in a climate-controlled electrospinning cabinet (IME Technologies, Geldrop, The Netherlands) according to the electrospinning method described previously by Pabittei et al. (23). Films were prepared by dissolving PC-UPy in HFIP, followed by casting the solution in a petri dish and dried at 37°C in a vacuum oven. Scaffolds and films were gamma sterilized.

### *In vitro* Degradation

#### PCL-Bisurea Scaffolds

Samples for the *in vitro* study were degraded following methods previously described by Brugmans et al. (10). In brief, circles with a diameter of 8 mm were punched from the electrospun material (*n* = 3 per treatment). Prior to the degradation process, meshes were centrifuged at 4,500 rpm in purified water for 10 min to wetten the material and remove air bubbles. The material was enzymatically degraded by incubation at 37°C in 0.5 mL enzyme for either 12 or 36 h. The enzyme solution consisted of a 100x dilution of 10 U/mL cholesterol esterase from bovine pancreas (C-3766, Sigma–Aldrich, Saint Louis, US) in PBS. Cholesterol esterase is present in native serum, secreted by activated macrophages and known to be more dominant for the cleavage of urethane bonds than other secreted enzymes (24, 25). Oxidative degradation was done in a 2 mL oxidative degradation solution. The oxidative solution consisted of 20% hydrogen peroxide (#216763, Sigma–Aldrich) and 0.033 M cobalt(II) chloride (#232696, Sigma–Aldrich) in purified water. Hydrogen peroxide and cobalt(II) chloride undergo a Haber–Weiss reaction, creating reactive hydroxyl radicals, which react with the material (26). Incubation times of the scaffolds in oxidative solutions were 2.5 and 5 days. As control, samples were exposed to PBS for 12 and 36 h. The oxidative solutions were changed every 2.5 days to maintain a constant concentration of radicals.

#### PC-UPy Films and Scaffolds

Scaffolds were punched to 25 x 5 mm strips (*n* = 4 per timepoint). Films were cut to 1 x 1 cm samples (*n* = 1). Scaffolds were centrifuged at 4,500 rpm in purified water for 3 min. The scaffold and film were degraded using oxidative degradation with 50 wt% H_2_O_2_ (Honeywell) at 50°C for 2, 5 and 20 days. The oxidative solutions were changed every 3 days to maintain a constant concentration of radicals. The reaction was stopped by washing the samples with RO water and 1.3 M Na_2_SO_3_ solution.

### Degradation Analysis

For routine degradation analysis, the mass averaged molecular weight (M_w_) of three samples per condition were determined, by dissolving them in dimethylformamide (DMF) (Sigma-Aldrich) followed by gel permeabilization chromatography (GPC) analysis. This analysis was performed on a Varian/Polymer Laboratories PL-GPC 50, using DMF with 10 mmol/L lithium bromide as eluent and maintaining the temperature of the equipment at 50 °C. Furthermore, scaffold fiber morphology was visualized with scanning electron microscopy (SEM, Quanta 600F; Fei, Hillsboro, OR) to assess scaffold damage after *in vitro* degradation.

### *In vivo* Degradation

Tissue sections of PC-UPy grafts were obtained from an *in vivo* study of a carotid implant in a sheep model (27). The animal study was conducted in compliance with ethical regulations and the Helsinki protocol. Furthermore, animal welfare was in compliance with the Directive 2010/63/ EU. PC-UPy scaffolds were implanted in 6 female Ile de France sheep (age 8–9 months, weight 45–90 kg at implantation) in the interposition of the carotid artery at IMMR (Paris, France). All animals received a 6–7 mm inner diameter, 3–5 cm long PC-UPy graft. The follow-up was 6 (*n* = 3) and 12 months (*n* = 3). All animals received Enoxaparin treatment (3000–4000IU SC/BID) until day 90 and Aspirin (125 mg IM/QD) until sacrifice. Patency was 100% at 6 months (*n* = 3) and 12 months (*n* = 3) follow-up with no indications of stenosis. After the endpoint of the study the explants were fixed in 4% neutral buffered formalin. The specimens were dehydrated, embedded in paraffin, and sectioned at 4 to 10 μm (performed by CVPath Institute Inc., Gaithersburg, US). For orientation in the section and to define relevant regions for Raman imaging, overview staining *via* Movat Pentachrome were provided as well as von Kossa stains to detect calcifications (Supplementary Figure 1). Based on histochemical images, histo-scores were defined for semi-quantification of matrix degradation and collagen infiltration as previously described by Brugmans et al. (28). Histo-scores for polymer absorption, collagen infiltration and calcifications as well as the definition of score values are described in Supplementary Figure 2.

### Raman Microspectroscopy

An inverse Raman microspectrometer (alpha 300R, WITec GmbH, Ulm, Germany), equipped with a 532 nm laser diode, was employed for all measurements with laser output power set to 50 mW for spectral acquisition (600 gr/mm, ±10 wavelengths).

Measurements of *in vitro* degraded scaffolds were performed using a 50x objective (LD EC Epiplan-Neofluor HD DIC 50x/0.55, Zeiss, Oberkochen, Germany). The integration time per spectrum was set to 0.2 s. For each sample, a minimum of three ROIs was chosen based on bright field images. Dimensions of the selected ROIs were 100 x 100 μm at a spatial resolution of 1 x 1 μm.

**Figure 1:**
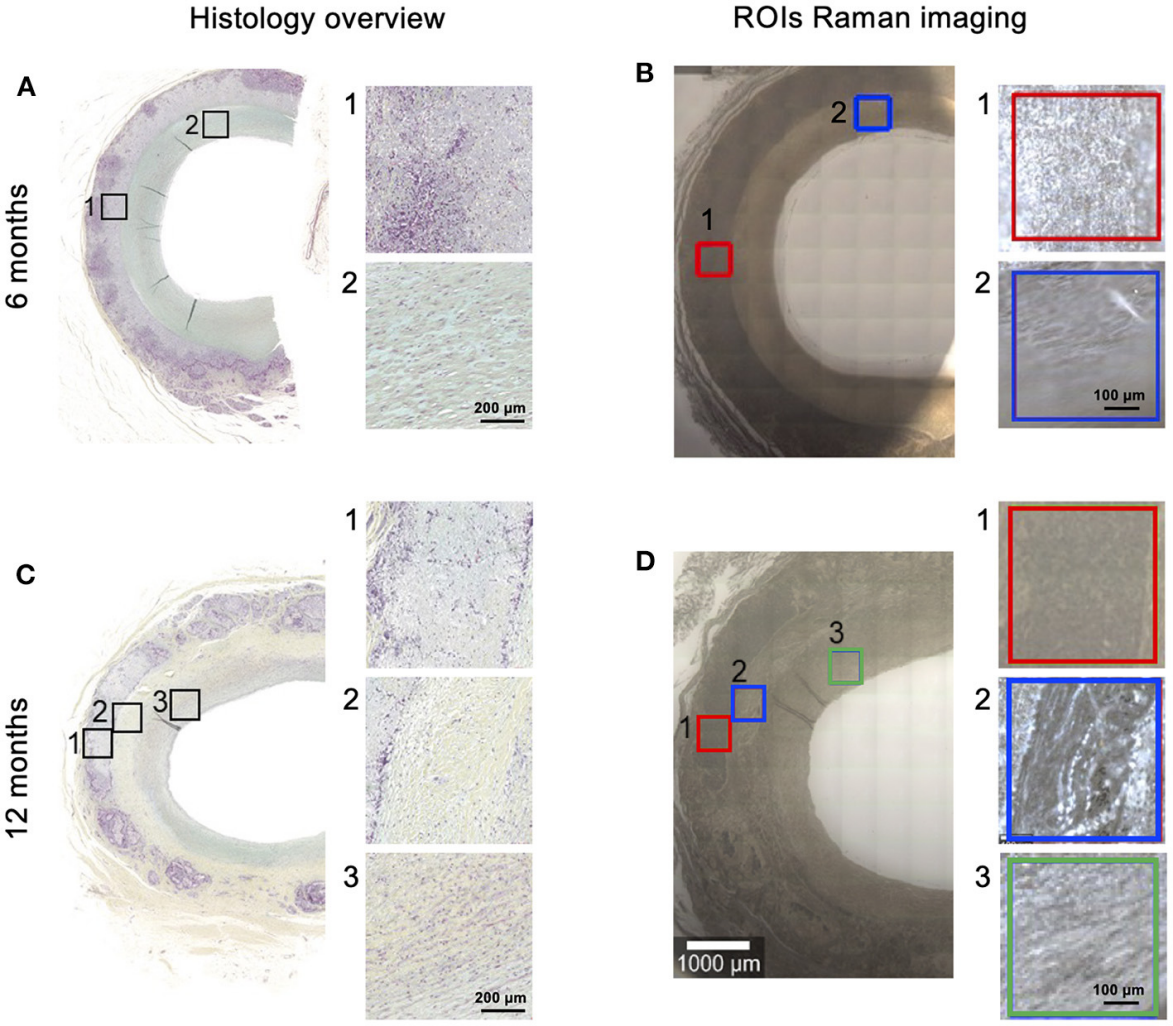
Overview of analyzed scaffolds and regions of interest (ROIs). PC-UPy scaffolds were implanted in a sheep model. FFPE sections of 6-month **(A,B)** and 12-month **(C,D)** explants were analyzed. ROIs for Raman imaging **(B,D)** were defined in Movat-Pentachrome stained sections **(A,C)** (provided by CVPath). In 6-month samples scaffold material (1) and lumen (2) were distinguished, in 12-month samples scaffold material (1), material-tissue interface (2) and neointimal lumen (3) were defined as ROIs.

All measurements of *in vivo* degraded samples were performed with a 63x water-immersion objective (W Plan Apochromat 63x/1, Zeiss). Prior to Raman measurements, the tissue sections were deparaffinized and kept hydrated in PBS. Raman images were acquired from defined regions (500 x 500 μm, 2 x 2 μm/pixel, 0.05 s/pixel) within the implanted material and neointima, as well as the material-luminal tissue interface for the 12 months samples (Figure 1). ROIs were defined in histology overview images (Movat-Pentachrome stain performed by CVPath Institute Inc., Gaithersburg, US) and transferred to unstained sections for Raman imaging. To avoid processing artifacts (e.g. by deparaffinization), *in vitro* degraded PC-UPy reference materials (see section *In vitro* Degradation) were additionally embedded, sectioned and deparaffinized before Raman imaging at the same conditions as the tissue sections to allow a direct comparison.

For spectral processing, *WITec Project FIVE (5.3)* software was used. Raman data was preprocessed by employing cosmic ray removal, cropping the spectra to the range between 300 and 3000 cm^−1^, baseline-correction (shape algorithm) and normalization (Area under the curve = 1). Intensity distribution heatmaps at 1444 cm^−1^ were generated from non-normalized *in vitro* PC-UPy degradation data and compared by mean gray value analysis in *ImageJ* (Fiji).

### Data Analysis

#### True Component Analysis

Processed images were analyzed using True Component Analysis (TCA), which aims to demix the different components in the measured spectra to find individual spectral components within the spectral dataset. Thus, it enables to generate false-color coded intensity distribution heatmaps based on different underlaying spectral information in each pixel of the spectral maps. Spectra of non-degraded control material were used as reference.

#### Principal Component Analysis

Single spectra of the scaffold polymer component as well as the collagen component were exported using *Project FIVE* software by generating an image filter of the TCA heatmaps. From each sample, a minimum of 100 spectra were randomly selected and exported to The Unscrambler X 10.3 (CAMO Software, Oslo, Norway). Principal component analysis (PCA) models were calculated for in-depth comparison of RM datasets of all conditions. PCA enables extraction of vector-dependent score values and loading spectra, which describe the spectral variances of a data set and depict the irregularities in a single Raman spectrum (29). PCA was performed employing the non-linear iterative partial least squares algorithm as described previously (30). All principal components (PCs) were screened for changes in Raman shifts found in the fingerprint regions of Raman spectra of the scaffold polymer or collagens. Outliers in PCA were determined using Hotelling T^2^ test and excluded.

### Statistical Analysis

Statistical analysis was performed in Graph Pad Prism 9. Data are presented as mean ± standard deviation. Data were tested for Gaussian distribution (Shapiro-Wilk) and statistically compared using one-way ANOVA and Tukey test or Kruskal-Wallis and Dunn's test for multiple comparisons. *P* values < 0.05 were considered statistically significant.

## Results

### Polymer Scaffolds Exhibit Characteristic Raman Signatures

**Figure 2:**
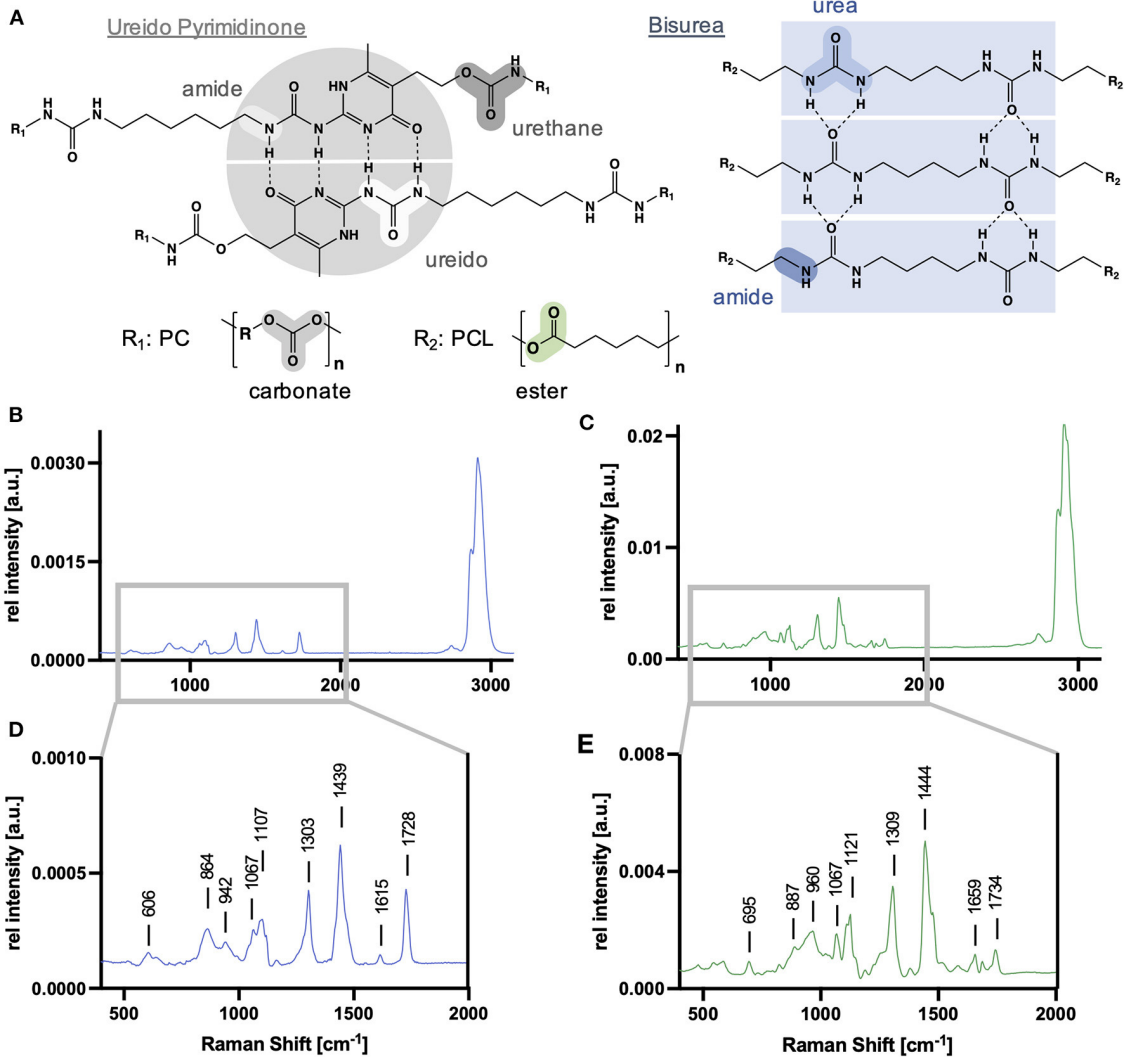
Supramolecular building blocks and Raman spectra of scaffold polymer references. **(A)** Generic molecular structure of ureido pyrimidine polycarbonate (PC-UPy) and bisurea polycaprolactone (PCL-BU) including their molecular groups susceptible to degradation. PCL-BU **(B,D)** and PC-UPy **(C,E)** exhibit characteristic Raman signatures with bands assigned to their different building blocks.

Raman spectra of non-degraded electrospun PCL-BU and PC-UPy (Figure 2) were acquired and served as references to identify degradation-dependent spectral changes. Generic molecular structures for UPy and BU building blocks and relevant functional groups susceptible to degradation are described in Figure 2A. For PCL-BU (Figures 2B,D), a distinction could be made between the fingerprint regions resulting from the PCL chains and the urea blocks. When considering the PCL bands, peaks assigned to the C-C vibrations of the C-COO group (862 and 942 cm^−1^), skeletal stretching (1,067 and 1,098 cm^−1^), deformation vibrations of the CH_2_ groups (1,303 and 1,442 cm^−1^) and vibrations caused by C=O vibration (1,728 cm^−1^) were identified (31). The fingerprint regions of the urea can be assigned to NCO and NCN vibrations (606 cm^−1^) and N-H stretching at 1,615 cm^−1^ (32). The Raman spectrum of PC-UPy (Figures 2C,E) demonstrates assignments to the UPy blocks especially at 695 and 1,659 cm^−1^. Spectral resonance typical for the PC chains is reflected by C-COO (960 cm^−1^) and C=O groups (1,734 cm^−1^), skeletal stretching (1,067 cm^−1^) as well as CH_2_ deformations (1,309 and 1,444 cm^−1^). An overview of the most relevant bands and their molecular assignments is given in Table 1.

**Table 1:** Relevant PCL-BU and PC-UPy peaks and their molecular assignments.

**Raman Shift [cm** ^**−1**^ **]**		**Molecular assignment**
**PCL-BU**	**PC-UPy**	
606		N-C-O and N-C-N vibrations (BU)
	695	Heterocycle ring vibrations (Upy)
864	887	C-O-C stretch
942	960	C=O
1067	1067	C-C bending (polymer backbone)
1107		C-C stretch (PCL)
	1121	C-C stretch or C-OH/C-O-C
1303	1309	CH_2_ twisting
1439	1444	CH_2_/CH_3_ bending
	1520	C=C
1615		N-H
	1659	UPy
	1685	Amide (urethane)
1728		C=O
	1734	C=O (PC)

### Comparison of Oxidative and Enzymatic *in vitro* Degradation in PCL-BU Scaffolds

**Figure 3:**
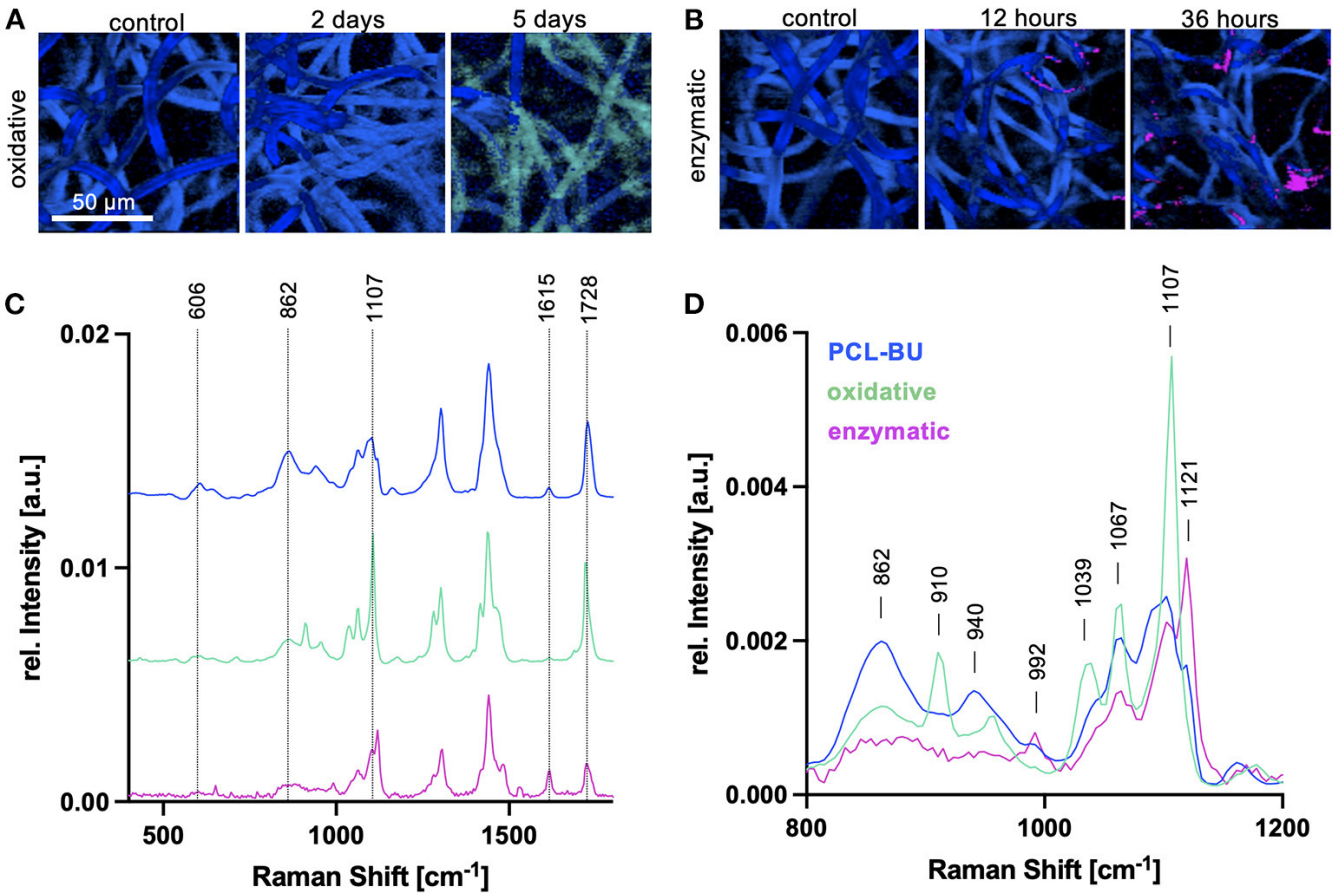
Oxidative and enzymatic degradation in PCL-BU scaffolds. **(A)** TCA heatmaps of electrospun PCL-BU scaffolds at 0, 2 and 5 days of oxidative degradation. Scale bar equals 50 μm **(B)** TCA heatmaps of electrospun PCL-BU scaffolds at 0, 12 and 36 h of enzymatic degradation. **(C)** Average Raman spectra identified by TCA. blue—PCL-BU; light green—signature in 5d oxidatively degraded PCL-BU fibers; pink—signature appearing after 12 h of enzymatically degraded PCL-BU fibers. Labeled peaks correspond to bands with changes in peak intensity. **(D)** Zoom into the 800–1,200 cm^−1^ Raman shift range.

First, the effect of different degradation mechanisms was investigated on PCL-BU material. PCL-BU was selected as it was previously reported that this material is susceptible to both enzymatic and oxidative degradation (10). Electrospun PCL-BU scaffolds underwent oxidative degradation using H_2_O_2_/CoCl_2_ or enzymatic degradation by cholesterol esterase. Raman images were acquired at three different timepoints for each of the degradation procedures and analyzed by True Component Analysis (TCA) to generate intensity distribution heatmaps by fitting the non-degraded PCL-BU reference spectrum. In oxidative degradation, TCA of PCL-BU scaffolds allowed to visualize the fiber structures with no significant changes in fiber morphology over degradation time. However, for the 5 day degraded scaffolds, there was no good fit with the PCL-BU reference spectrum and an additional TCA component (light green) was identified (Figure 3A). TCA with the PCL-BU reference spectrum was also applied to generate Raman images of the enzymatically degraded scaffolds. Here, a clear change in fiber morphology to less and also thinner polymer fibers was observed (Figure 3B). Moreover, TCA identified a new spectral component (pink), starting to appear after 12 h of degradation at a spot wise localization. The two new spectral components were compared to the PCL-BU reference spectrum and showed changes in signal intensities in the Raman peaks at 606, 862, 1,107, 1,615 and 1,728 cm^−1^ (Figure 3C) but also in the overall spectral pattern, especially in the wavenumber range between 800 and 1,200 cm^−1^ (Figure 3D). New peaks which were either just present as spectral shoulder of another band or not expressed at all in the non-degraded PCL-BU spectrum were identified, i.e. at 910 and 1,039 cm^−1^ upon oxidative degradation and 992 and 1,121 cm^−1^ upon enzymatic degradation.

### Monitoring of Oxidative *in vitro* Degradation in PC-UPy Scaffolds

**Figure 4:**
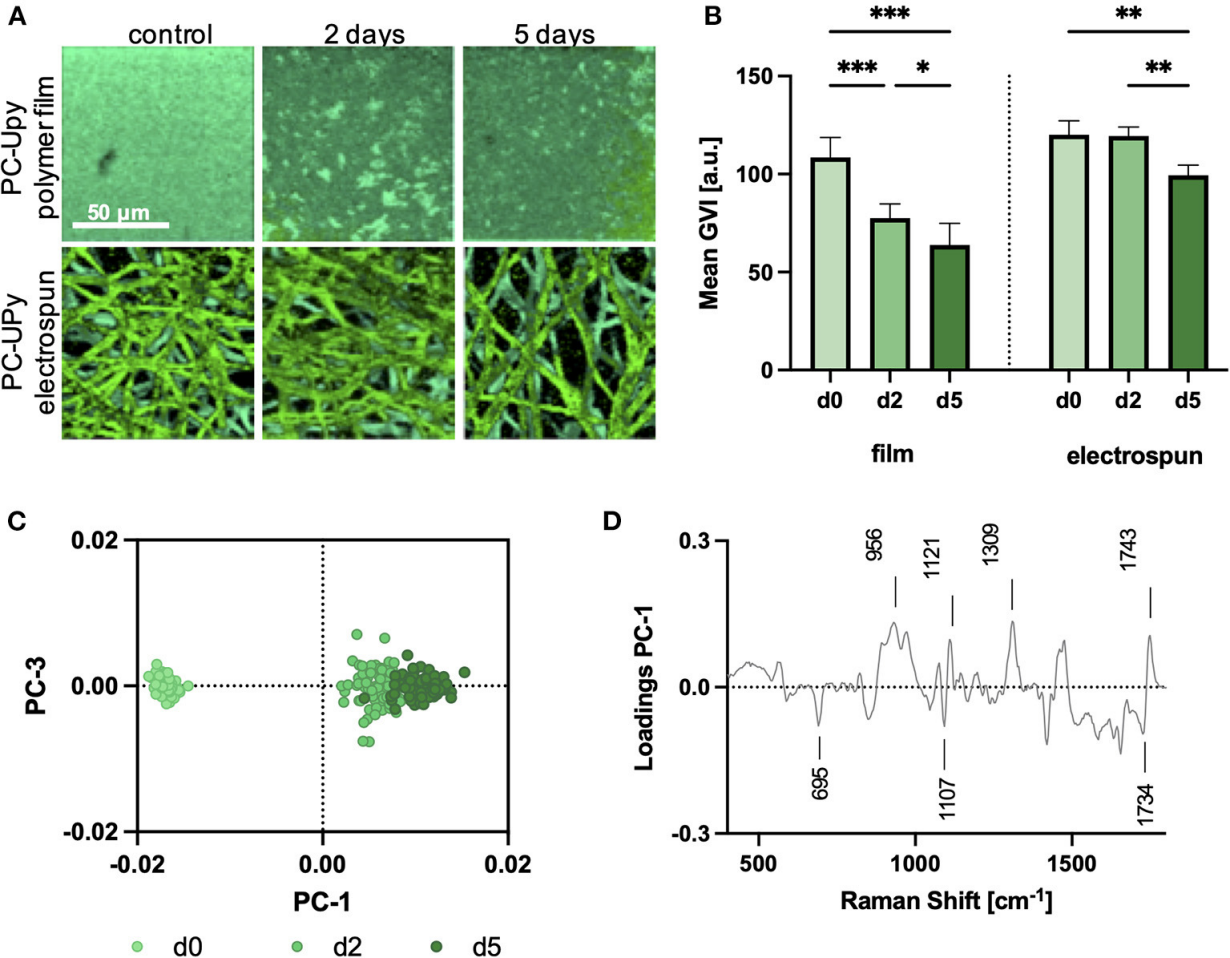
Comparison of oxidative *in vitro* degradation of film and electrospun PC-UPy polymers. **(A)** TCA images of polymer films (upper row) and electrospun scaffolds (lower row) at 0 (d0), 2 (d2) and 5 (d5) days of oxidative *in vitro* degradation. Scale bar equals 50 μm **(B)** Mean gray value intensities (GVI) of the PC-UPy polymer film and electrospun fiber intensity distribution heatmaps. Data are represented as mean ±SD; *n* = 3, one-way ANOVA, *p < 0.05, **p < 0.01, ***p < 0.001. **(C)** PC-1/PC-3 scores plot of a PCA performed on single extracted spectra from electrospun fibers **(A)** demonstrates a degradation time-dependent shift in the Raman signature. **(D)** The PC-1 loadings plot indicates relevant peaks for the spectral separation.

Raman microspectroscopy was then performed on *in vitro* degraded PC-UPy polymer films and electrospun scaffolds to determine the sensitivity of Raman microspectroscopy to investigate polymer degradation (Figure 4). Given that PC-UPy is barely susceptible to enzymatic degradation *in vitro* (33), the samples for this material were degraded by exposure to H_2_O_2_. TCA, with the non-degraded PC-UPy as reference spectrum, allowed for the mapping of the non-degraded material within the samples (Figure 4A). Control PC-UPy films showed a homogenous distribution of the Raman signal which decreased after 2 and 5 days within the degradation solution as quantified by the mean gray value intensity (GVI, Figure 4B). TCA images of electrospun control and degraded PC-UPy scaffolds expressed a good fit to the PC-UPy reference spectrum and showed tendencies toward a less dense fiber network after 5 days, as also reflected in the mean GVI (Figure 4B) but no further morphological differences. Therefore, a defined number of single spectra were randomly extracted from the TCA maps and analyzed by principal component analysis (PCA) to determine minor spectral changes and investigate the molecular composition of the degraded samples. The PC-1/PC-3 scores plot showed a clear separation of the spectral information derived from non-degraded, but also between day-2 and day-5 degraded polymer fibers. Control spectra clustered in the negative PC-1 scores range, whereas degraded samples clustered in the positive PC-1 range, with the strongest shift in day-5 scaffolds (Figure 4C). The PC-1 loadings plot served to interpret the molecular changes that are assigned to the observed degradation time dependent separation (Figure 4D). The further a data point clusters at the positive PC scores range, the more pronounced are the bands which are described as positive peaks in the loadings. Data clustering in the negative scores range, exhibit stronger assignments to bands which are negative peaks in the corresponding loadings plot. Here, the bands at 695 (UPy) and 1,107 cm^−1^ (C-C, skeletal stretch) decreased upon degradation, whereas an increase was observed for the bands at 956 (C=O), 1,121 (C-O-C, ether groups), 1,309 (CH_2_ twisting) and 1,743 cm^−1^ (C=O).

### Molecular Characterization of *in vivo* Degradation of PC-UPy Scaffolds

**Figure 5:**
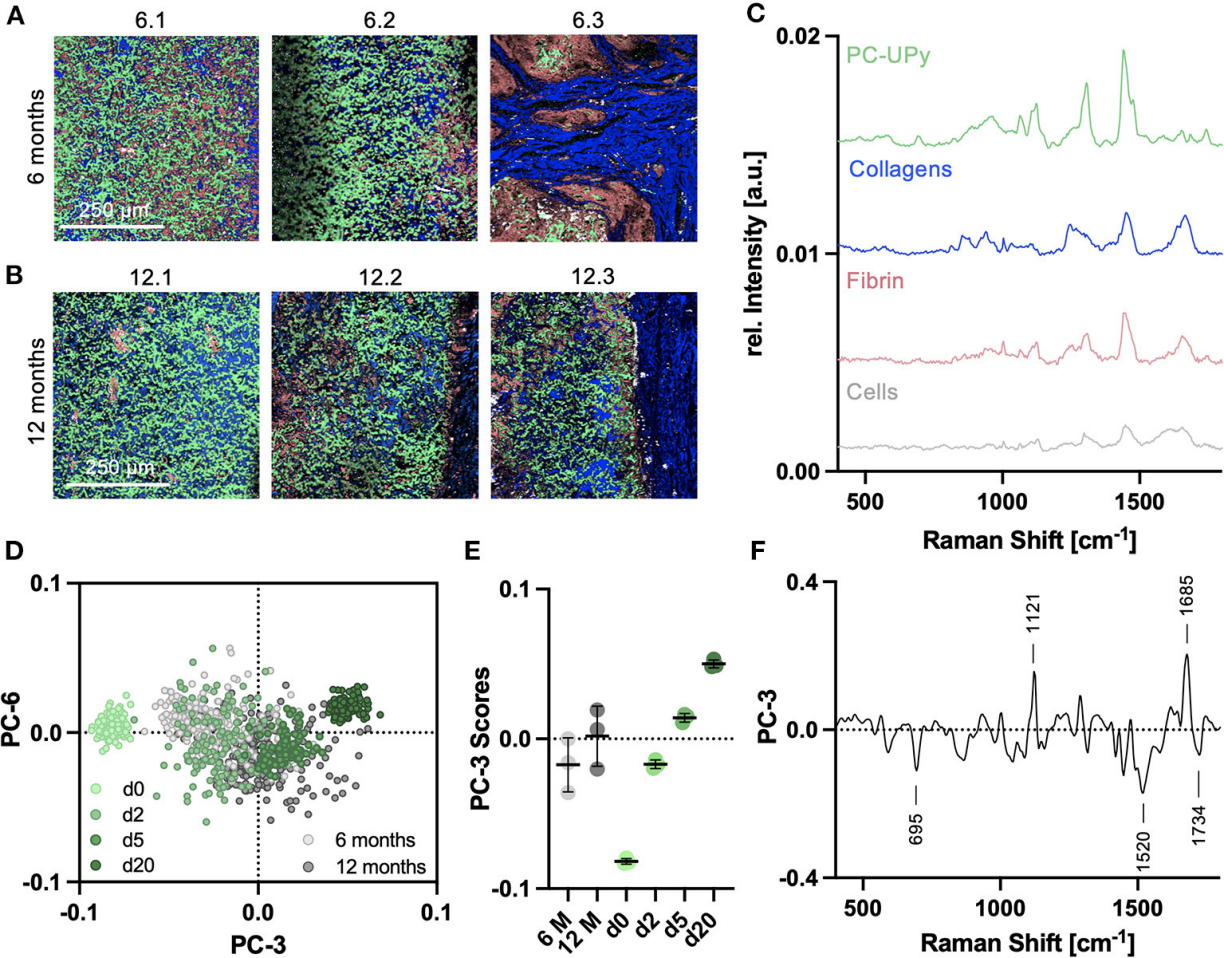
Raman analysis of *in vivo* degradation of PC-UPy scaffolds. TCA Raman images of **(A)** 6-month and **(B)** 12-month explants; light green—PC-UPy, blue—collagens, light red—fibrin, white—cells, each image represents the explant from another animal, scale bar equals 250 μm **(C)** Identified TCA Raman spectra could be assigned to polymer and biological signatures. **(D)** Spectra extracted from *in vivo* degradation were compared to *in vitro* degradation by principal component analysis. **(E)** PC-3 scores indicated a degradation time dependent shift with 6 and 12-months *in vivo* samples clustering in the range between 2 and 5 day *in vitro* degraded samples, *n* = 3, mean score values ± SD **(F)** Underlaying spectral changes are explained by the PC-3 loadings plot.

Tissue sections of an *in vivo* study, where PC-UPy based electrospun scaffolds were implanted as carotid replacement in a sheep model, were investigated by Raman imaging. Spectral maps of the scaffold region (ROI1) of 6-month (Figure 5A) and 12-month explants (Figure 5B) were analyzed by TCA. In addition to a strong signal originating from the PC-UPy polymer, TCA allowed to localize and identify collagens, cells and fibrin depositions according to characteristic spectral signatures (Figure 5C). Whereas two of the 6-month explants showed a major contribution of the polymer fibers, in one explant (6.3) a strong collagen ingrowth was observed. Similarly, one of the 12-month implants (12.1) resulted in strong PC-UPy contributions compared to the other two 12-month explants, where the polymer assignments appeared less dense. Even though differences were visible between 6- and 12- month image distributions, 12-month explants still showed major assignments to polymer materials. Semi-quantitative analysis of polymer resorption was performed and defined in Histo-Scores, indicating a score of 25-50% matric absorption after 12 months (Supplementary Figure 2). In addition to morphological differences, spectra from the TCA images were extracted and analyzed by PCA to investigate molecular changes in PC-UPy polymer fibers upon *in vivo* degradation and to compare them to *in vitro* degradation. Therefore, a PCA including spectra from *in vitro* as well as *in vivo* degraded scaffolds was conducted. *In vitro* data were extended by an additional timepoint after 20 days of oxidative degradation. The PC scores plot demonstrated a degradation time dependent shift toward increasing PC-3 score values with progressing degradation time and showed a close overlay of *in vivo* and *in vitro* degraded samples (Figure 5D). Comparison of the PC score values (Figure 5E) demonstrated a clustering of the 6-month samples within the range of the 2-day *in vitro* degraded samples, whereas 12-month samples were overlapping with the 5-day degraded samples. In accordance with the TCA images, the signatures of the 12.1. explant resembled rather the composition of the 6-month explants. An explant-wise comparison including an overview of the statistical testing is provided in Supplementary Figure 3. The PC-3 loadings plot (Figure 5F) allows to interpret the underlaying molecular changes. Polymer fibers at later degradation stages, clustering at higher PC-3 ranges, were assigned to more pronounced bands at 1,121 (C-O) and 1,685 cm^−1^ (Amide, urethane), whereas signatures of non-degraded and early-stage degraded samples, clustering in the negative PC-3 range, had a higher contribution of the peaks at 695 and 1520 cm^−1^ (both UPy).

### *In vivo* Tissue Remodeling Correlates to Polymer Degradation

**Figure 6:**
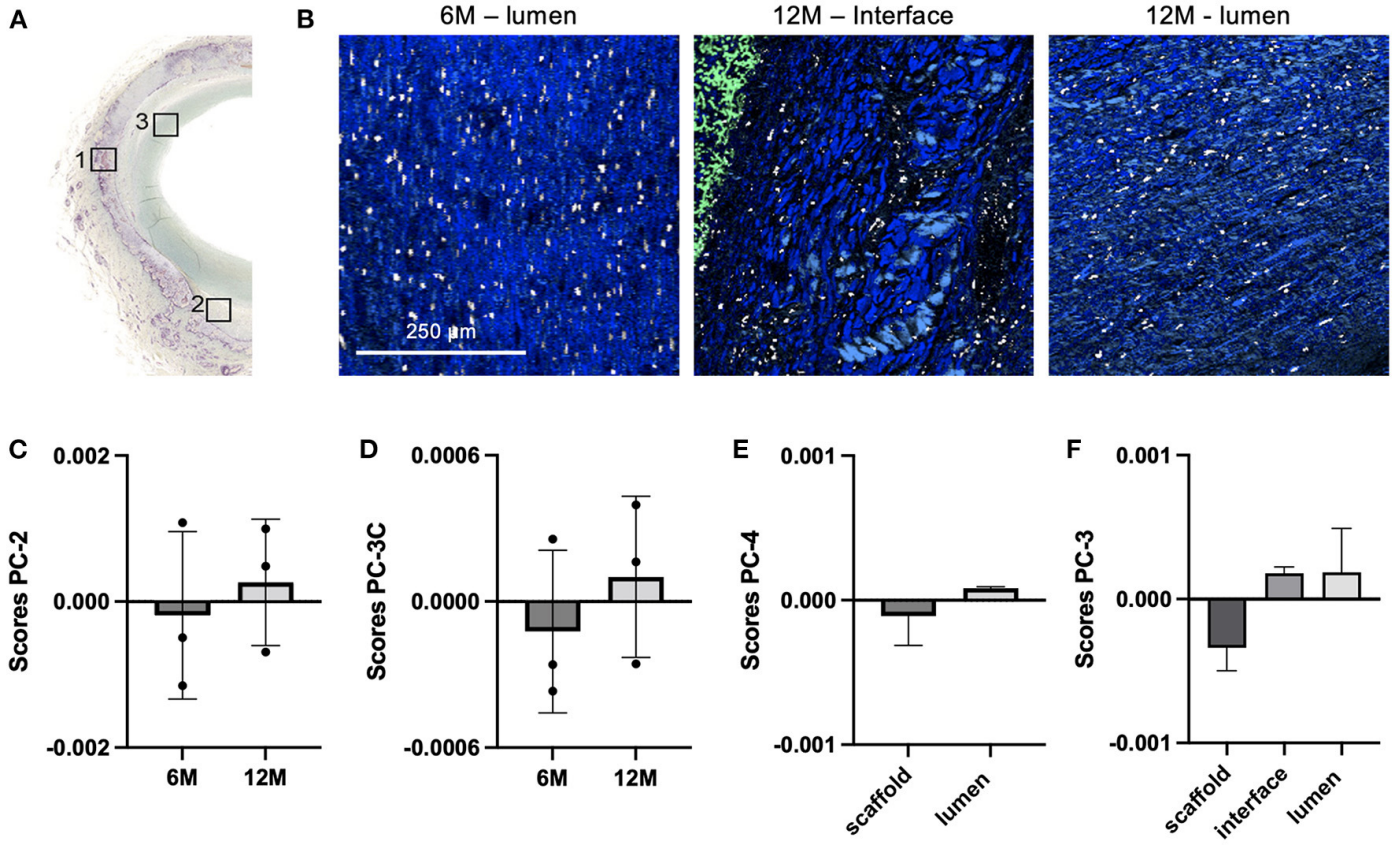
Tissue maturation and collagen infiltration in carotid grafts. **(A)** Raman images were acquired from additional regions at the explant lumen (ROI3) and the interface (ROI2, only in 12-month samples), as defined in consecutive sections stained for Movat Pentachrome. **(B)** TCA intensity distribution heatmaps including collagen (blue), PC-UPy (light green) and cells (white) signatures; scale bar equals 250 μm. Extracted collagen spectra were compared by PCA for **(C)** 6 vs. 12-month scaffold region (ROI1), **(D)** 6 vs. 12-month luminal region (ROI3), **(E)** scaffold vs. luminal region in 6-month samples and **(F)** scaffold vs. interface vs. luminal region in 12-month samples; data are represented as mean score values ± SD, *n* = 3.

Raman histopathology enables the simultaneous detection and analysis of both, polymer and biological components, within the same tissue section and without the need for extensive sample processing or staining. In addition to polymer degradation at the implant material, the surrounding tissue was analyzed at three different regions—in between the scaffold fibers, at the polymer-tissue interface and in the neointima at the lumen (Figure 6A). The three-layer separation was only significantly expressed in the 12-month explants, whereas the 6-month explants were separated to the scaffold site and the luminal tissue layer. In one of the 12-month samples (12.1) it was only possible to distinguish scaffold and lumen region (Supplementary Figure 4). The major extracellular matrix component identified in the adjacent tissue layers were collagen fibers as indicated in the TCA Raman images (Figure 6B). Single spectra were extracted from the TCA images for further in-depth analysis of collagen fiber maturity and integrity. PCAs were performed comparing collagen signatures of the same ROI at different timepoints and among different ROIs for the same timepoint. PCA of collagens infiltrating the scaffold region (Figure 6C) showed a shift from negative PC score values in 6-month spectra to positive PC score values in 12-month samples. However, both groups demonstrated data from one animal (6.3 and 12.1) clustering rather with the data from the other timepoint. A similar trend was shown for the collagens of the luminal tissue layer after 6 and 12 months of implantation (Figure 6D). The corresponding loadings plots (Supplementary Figures 5A,B) describe relevant peaks that explain the implantation time dependent changes in collagen fibers between 6 and 12 months. Both loadings indicate similar molecular changes in the scaffold and luminal region that are assigned to increased bands at 853, 935 (proline), 1,244 (amide III, ß-sheet), 1,422 (CH_2_ deformation) and 1,639 cm^−1^ (amide I) in 12-month explants and increased bands at 1,003 (phenylalanine), 1,310 (amide III, α-helix) and 1,690 cm^−1^ (amide I) for 6-month data. Separate PCAs were performed to compare collagens of the scaffold and luminal region at 6 months (Figure 6E) as well as collagens from scaffold, interface and luminal region at 12 months after implantation (Figure 6F). Six-month data clustered closely together with no significant difference between the regions for any of the calculated PCs, whereas 12-month data demonstrated a shift between collagen signatures from the implant region to collagen spectra derived from the interface and luminal region. However, also here no statistical significance was reached due to the heterogeneity among the animals. Peaks that contributed to the spectral differences were elaborated by the corresponding PC loadings plot (Supplementary Figures 5C,D) and could be assigned to differences in proline (853 cm^−1^), amide III (1,240 cm^−1^), the collagen backbone (960 and 1,440 cm^−1^) and the amide I conformation (1,660 cm^−1^).

## Discussion

Understanding the degradation of implants prospering from the recipient's endogenous regenerative capacity *via in situ* tissue engineering is of high relevance for the design and success of novel implant devices for cardiovascular replacements. In this study, Raman microspectroscopy was proposed as a complementary method to conventional analyses such as SEM or GPC analyses to study and evaluate local polymer degradation, as it allows to access information on the molecular composition and can be performed without extensive sample processing, i.e. on tissue sections which are already prepared for histological analyses. Compared to destructive techniques such as chromatographic, mechanical or thermal analyses which have been reported for the investigation of *in vivo* polymer degradation (34, 35) and rather allow to assess the overall condition and characteristics of an implant material, Raman imaging obtains molecular-sensitive and spatially resolved information on the sample composition.

We have previously reported on the use of Raman microspectroscopy as a complementary method to evaluate the local balance between scaffold degradation and neotissue formation in *in situ* tissue-engineered heart valves (15) and arteries (5). In the current study, we specifically investigated whether the modus of degradation (i.e. enzymatic or oxidative) could be distilled from *in vivo* samples. To that end, Raman microspectroscopy was implemented to investigate different aspects of *in vitro* degradation such as the influence of the selected polymer, degradation over several timepoints as well as oxidative and enzymatically induced degradation. To further study the effect in native tissue, material degradation was monitored using Raman imaging on carotid explants of an *in vivo* sheep study.

Two different polymers were investigated for the *in vitro* study to assess the robustness of Raman spectroscopy to investigate degradation throughout different polymer materials. The two supramolecular polymer composites PC-UPy and PCL-BU were applied as model substances, as they are potential candidates for tissue engineering approaches (10, 36, 37) and provide different degradation kinetics. In general, PC-based polymers have been shown to degrade slower than PCL-based polymers (38). Despite similarities arising from similar molecular groups and assignments to skeletal vibrations of the polymer backbone, the Raman signatures of both polymers were significantly different, which in the future would also allow to discriminate oxidative processes in various other polymer materials applied in tissue engineering and simultaneously analyze implants composed of more than just one polymer.

Raman imaging of the degraded scaffolds allowed for the non-destructive visualization of the fiber morphology. Unlike SEM, which can only assess the fiber morphology, Raman spectroscopy allows for molecular imaging, investigating the molecular composition of the sample. Effects of oxidative degradation in PC-UPy polymers were less pronounced than in PCL-BU scaffolds but are in accordance with the slower degradation kinetics of PC-based polymers (38). Whereas changes in molecular composition upon PC-UPy degradation were only detectable *via* in-depth multivariate comparison by PCA, degradation in PCL-BU was already reflected in TCA images with a significantly different spectral signature of the polymer fibers after 5-day oxidative degradation. These data correlate with findings from an *in vivo* study of PCL-BU-based carotid implants in a rat model which showed highly accelerated degradation (39).

Degradation mechanisms for UPy and PCL based materials have been reported to be rather oxidative than hydrolytic (10, 33), therefore the *in vitro* degradation mainly focused on oxidative processes. However, an enzymatic degradation step *via* cholesterol esterase was included for PCL-BU and compared to oxidative degradation. In addition to differences in fiber morphology, TCA images identified degradation-dependent spectral signatures which demonstrated different distribution patterns between oxidative and enzymatic degradation. The hypothesis here is that in enzymatic degradation entire molecules are cleaved, causing surface erosion which will decrease the fiber diameter and identify degradation-specific spectral changes at local spots. In contrast, the molecules in the oxidatively treated samples are believed to remain in place, while fiber length is reduced, which will not affect the fiber diameter but the overall molecular composition and spectral signature of the fibers. These conclusions are further supported by conventional degradation analyses by SEM and GPC (Supplementary Figure 6), where changes in fiber morphology are more pronounced in enzymatic degradation, but fiber density and composition, reflected by changes in the molecular weight, is rather observed in oxidative degradation. Furthermore, different molecular assignments of the TCA components appeared upon degradation. Compared to non-degraded PCL-BU, the spectrum in oxidatively degraded scaffolds showed a shift of the intensity ratios of the different building blocks. BU-related peaks (606, 1,615 cm^−1^) decreased, whereas bands assigned to the skeletal vibrations and PCL ester groups (1,067, 1,107, 1,728 cm^−1^) increased. This phenomenon can be assigned to relative changes in the polymer composition, as oxidative processes degrade the bisurea bonds first, resulting in higher contributions of the PCL-related bands to the overall polymer signature. Additional peaks occurring at 910 and 1,039 cm^−1^ could potentially be assigned to newly formed carboxyl groups derived from ester or urethane moieties and have been reported in correlation with a relative increase in crystallinity observed upon PCL degradation, which starts at the amorphous regions of the polymer (40). The degradation-specific spectrum in enzymatic degradation showed opposite spectral shifts. Here, a strong decrease of PCL-related bands, especially carbonyl (1,728 cm^−1^) and ester (865 cm^−1^) groups, was observed as well as a relative increase of the N-H band (1,615 cm^−1^) and the shoulders at 992 and 1,121 cm^−1^. This signature is fully in line with ester hydrolysis taking place in the PCL-moieties of the fibers.

Following *in vitro* degradation, *in vivo* degradation of PC-UPy was assessed in 6 and 12-month carotid explants from a sheep model. Direct comparison to spectra from *in vitro* oxidative degradation supported the previous findings of Brugmans et al. (10) that oxidative mechanisms dominate in the degradation process for this material. Spectral signatures of 6-month *in vivo* data clustered closely with the 2-day *in vitro* degradation whereas 12-month spectra rather resembled the molecular composition of 5-day *in vitro* degraded samples. In addition to this overall trend, Raman spectroscopy also allowed to determine the individual degradational state for each explant. For each of the groups, an outlier explant was identified, rather clustering with the other explantation timepoint. Explant 6.3 showed an advanced degradation compared to the other 6-month samples, whereas spectra from explant 12.1 clustered closer to the 6-month or 2-day *in vitro* degraded samples. However, these observations are in line with the corresponding Raman and histology images, which show an advanced polymer degradation and collagen infiltration for explant 6.3 as well as a dense polymer network and no separation into a material interface and luminal layer for explant 12.1., supporting the sensitivity of Raman spectroscopy to characterize a sample's molecular composition.

Degradation-dependent spectral changes as defined in the loadings plots, were similar between *in vitro* and *in vivo* analyses. Most robustly, degradation was assigned to a decrease in UPy related bands (695, 1,520 and 1,734 cm^−1^) and an increase in the 1,121 cm^−1^ peak. The assignment of the 1,121 cm^−1^ peak has not been fully defined yet, but had already been reported in a previous study on degradation of a pulmonary valve implants (15). Moreover, the *in vivo* degraded samples were assigned to a more pronounced 1,685 cm^−1^ band which corresponds to the amide in urethane groups, potentially indicating a UPy degradation product. Overall, these results suggest that *in vivo*, PC-UPy degradation is dominantly induced by oxidative pathways to which the UPy bonds are more susceptible than the PC chains.

This study demonstrates the potential of Raman microspectroscopy and imaging to further advance *in situ* tissue engineering. Supramolecular polymers can be synthesized to customize topography, durability, strength and degradability of a graft (38, 41). Further insights on molecular degradation mechanisms that can be modeled and characterized *in vitro*, have been shown to serve as reference measurements that can be transferred to define *in vivo* degradation pathways and could allow to tailor and design polymer materials that form implants with a better *in vivo* longevity. In addition, the identification of degradation-time dependent spectral changes can serve as an *in vivo* readout to monitor and spatially resolve polymer degradation *in situ*. First studies for *in vivo* Raman spectroscopy have been realized through endoscopic setups (42, 43), and could be transferred in the future to assess the performance and degradation of *in situ* tissue-engineered implants marker-independently and *in vivo*.

The *in vivo* follow-up in the presented study monitored PC-UPy polymers over a timeline of 12 months, but not over the complete degradation period. Whereas PCL-BU grafts demonstrated highly accelerated degradation *in vivo*, resulting in scaffold absorption after 60 days in an arterial graft (39), PC-UPy scaffolds even did not show complete absorption after 2 years as a pulmonary valve graft in a sheep model (15). However, over time, neotissue was formed and initial inflammation was reduced (15, 28, 44), demonstrating more advanced absorption and the absence of a chronic inflammatory response or severe calcifications. These promising results paved the way for the initiation of first clinical trials for PC-UPy based cardiovascular grafts (45).

One additional important feature of Raman imaging is that it enables to simultaneously assess collagen infiltration and polymer degradation within the same region of a sample. The balance between polymer degradation and tissue deposition and maturation is very important for the outcome of *in situ* tissue engineering; too slow degradation may lead to chronic inflammation and fibrotic encapsulation, while too rapid degradation (i.e. before sufficient neotissue has formed to take over the mechanical loads), will lead to structural failure (46). Scaffold degradation *in vivo* is an active cellular process driven by inflammatory cells such as macrophages and foreign body giant cells, which also play a role in the coordination of tissue formation and maturation (47). This makes that scaffold degradation and tissue formation is dependent on the local influx and activation of cells and the local hemodynamic and biochemical environment, rather than a homogeneous time-dependent process. For example, Wissing et al. showed that macrophage-driven scaffold degradation is dependent on the scaffold microstructure (48) as well as hemodynamic loads (49). *In vivo*, Uiterwijk et al. observed a rapid unexpected accelerated remodeling of heart valve scaffolds with a bioinspired scaffold microstructure when implanted in sheep (50). Fukunishi et al. reported on species-dependent degradation rates of vascular grafts when implanted in rats or sheep (51). These reports emphasize the unpredictability of scaffold degradation and the tremendous value of accurately characterizing scaffold degradation and tissue formation simultaneously and *locally* in various regions of interest in a sample.

Therefore, in the present study, collagen spectra were extracted from the TCA images of the scaffold regions and compared to collagen signatures of newly formed tissue layers. Conventional methods to assess collagen deposition include histochemical or immunohistochemical staining (5) or can even be marker-independent when imaging collagen autofluorescence in SHG microscopy (4). In contrast to quantitative information, these methods are limited in their capability to address qualitative changes in structural composition of collagen fibers. Here, Raman spectroscopy has already been shown to be sensitive to changes in collagen fibers upon aging, tissue degradation or foreign body response (52–54) and was applied in this study to determine collagen maturation at different explantation timepoints and explant regions. Comparison between 6 and 12-month explants showed similar results in molecular changes for collagens between the polymer fibers and in the lumen. Upon implantation duration, collagen specific peaks assigned to proline, the collagen backbone and amides increased, whereas early-stage explants had a stronger phenylalanine peak assigned to proteins in general. Moreover, shifts in the amide I and amide III bands occurred which indicate different 2D and 3D structural orientation in α-helices and β-sheets, i.e. the prominent amide I shoulder at 1,690 cm^−1^ observed in 6-month samples can be linked to immature collagen crosslinks (55). Raman imaging of explant tissues served as a proof-of-principle experiment to study *in vivo* degradation, in combination with collagen maturation, and correlate it to observations of *in vitro* degradation. Sample heterogeneity was observed in this relatively small dataset in this study. Nevertheless, the ratios between polymer degradation and collagen infiltration and maturation were consistent throughout all samples, as the 6-month explant with faster polymer degradation kinetics simultaneously showed a more advanced collagen remodeling, same as for the slower degrading 12-month sample. These results encourage the further application and evaluation of PC-UPy scaffolds as carotid graft replacement.

## Conclusion

In this study, we have demonstrated the suitability of Raman microspectroscopy to investigate morphological and molecular changes during degradation of PCL- as well as PC-based supramolecular polymer composites. Raman imaging enables molecular sensitive *in situ* visualization of polymer degradation, overcoming the current limitations of conventional techniques. Characteristic peaks allowed to monitor degradation kinetics of different polymers and could distinguish oxidative and enzymatic degradation processes. Furthermore, partially degraded PC-UPy material could be identified and analyzed in *in vivo* degraded carotid implants, and was indicative of the predominant occurrence of oxidative degradation pathways. These findings underline the potential of Raman microspectroscopy and imaging to track the spatiotemporal progression of degradation and tissue maturation. The marker-independent accessibility of both, polymer and biological structures, open up a broad potential for *in situ* monitoring of tissue-engineered materials and will be further investigated in the future.

## Data Availability Statement

The raw data supporting the conclusions of this article will be made available by the authors, without undue reservation.

## Ethics Statement

The animal study was reviewed and approved by Ethics Committee n°37, CNREEA, France.

## Author Contributions

EM and EB performed Raman measurements. TW performed PCL-BU *in vitro* degradation experiments. JM analyzed and interpreted the data. HB, AS, AB, and SS validated the results. JM, HB, and AIS wrote the manuscript. KS-L, AS, AIS, and MC contributed to conceptualization, design and supervision of the study. All authors reviewed and edited the manuscript and approved the submitted version.

## Funding

The collaboration between Eindhoven University of Technology and the University of Tübingen for Raman microspectroscopy of PCL-BU was facilitated by a traveling grant of the Dutch Heart Foundation to EM and a Short-Term Fellowship (8169) of the European Molecular Biology Organization (EMBO) to AIPMS. This study was financially supported by the Ministry of Science, Research and the Arts of Baden-Württemberg (33-729.55-3/214 and SI-BW 01222-91 to KS-L) and the Deutsche Forschungsgemeinschaft (INST 2388/64-1 GRK 2543/1 and under Germany's Excellence Strategy EXC 2180-390900677 to KS-L).

## Conflict of Interest

The research labs from KS-L and AIS performed independent scientific contract work for the company Xeltis and received for this work financial compensation. MC, HB, and AS are employees of Xeltis. MC is shareholder of Xeltis and AB is a financially compensated scientific advisor to Xeltis. SS is an employee of SyMO-Chem BV. AB is an employee and shareholder in SupraPolix BV. The remaining authors declare that the research was conducted in the absence of any commercial or financial relationships that could be construed as a potential conflict of interest.

## Publisher's Note

All claims expressed in this article are solely those of the authors and do not necessarily represent those of their affiliated organizations, or those of the publisher, the editors and the reviewers. Any product that may be evaluated in this article, or claim that may be made by its manufacturer, is not guaranteed or endorsed by the publisher.
