# Supplementary material for: Marker-Independent Monitoring of in vitro and in vivo Degradation of Supramolecular Polymers Applied in Cardiovascular in situ Tissue Engineering
Source: Front Cardiovasc Med. 2022 May 17;9:885873. doi: 10.3389/fcvm.2022.885873 (PMC9152121; doi:10.3389/fcvm.2022.885873)
Supplement: Supplementary file 1 [file Data_Sheet_1.PDF]

Supplementary Material

1.1 Supplementary Figures

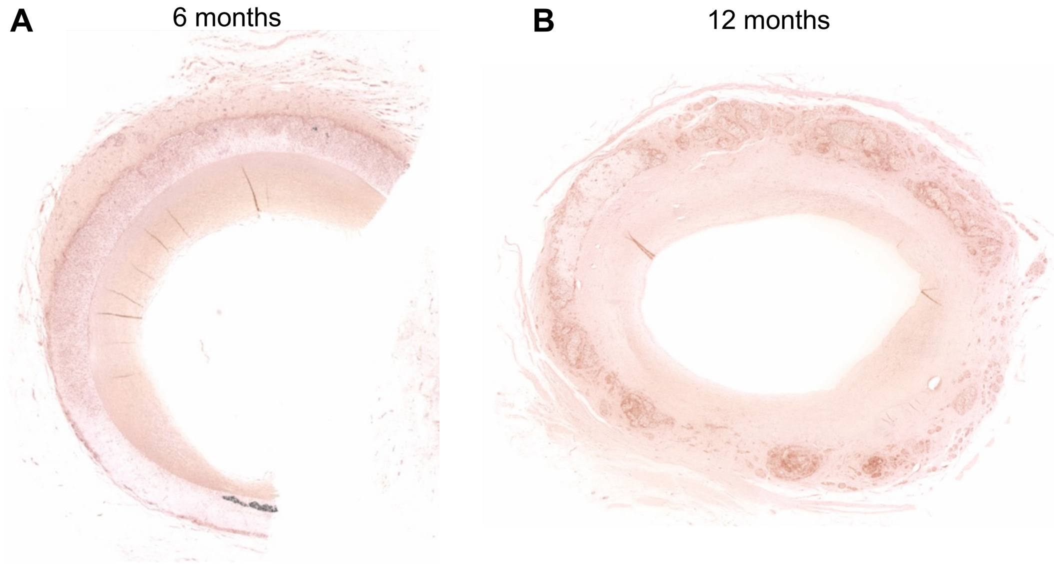

Supplementary figure S1. Histology of calcifications. Von Kossa staining of 6-month **(A)** and 12-month **(B)** explants did show no to minor signs of calcifications, limited to the suture region.

A) Histo-Scores (12 months)

	Collagen replacement	Matrix absorption	Calcification
**Prox. anastomosis**	2.3 ± 0.5	3.0 ± 0.8	1.7 ± 0.5
**Body**	3.0 ± 0.8	3.0 ± 0.8	1.3 ± 1.9
**Dis. anastomosis**	3.3 ± 0.9	3.3 ± 0.9	2.7 ± 0.9

B) Description of semi-quantitative scores

Attribute	Score	Description of Assigned Score
**Conduit matrix deposition**	**0**	No collagen replacement
	**1**	Collagen replacement <10% conduit length
	**2**	Collagen replacement 10 - 25% conduit length
	**3**	Collagen replacement 25% - 50% conduit length
	**4**	Collagen replacement 50% - 75% conduit length
	**5**	Collagen replacement ≥75% conduit length
**Calcification**	**0**	No calcification
	**1**	< 1% overall conduit area involvement
	**2**	1-5% overall conduit area involvement
	**3**	5-10% overall conduit area involvement
	**4**	> 10% overall conduit area involvement
**Matrix absorption score**	**0**	No absorption of matrix
	**1**	< 10% conduit length shows matrix absorption
	**2**	10-<25% conduit length shows matrix absorption
	**3**	25-<50% conduit length shows matrix absorption
	**4**	50-<75% conduit length shows matrix absorption
	**5**	>75% conduit length shows matrix absorption

Supplementary figure S2. Histo-Scores to define matrix absorption and collagen infiltration. (A) Overall histo scores of 12-month data. **(B)** Definition of semi-quantitative scores for each parameter.

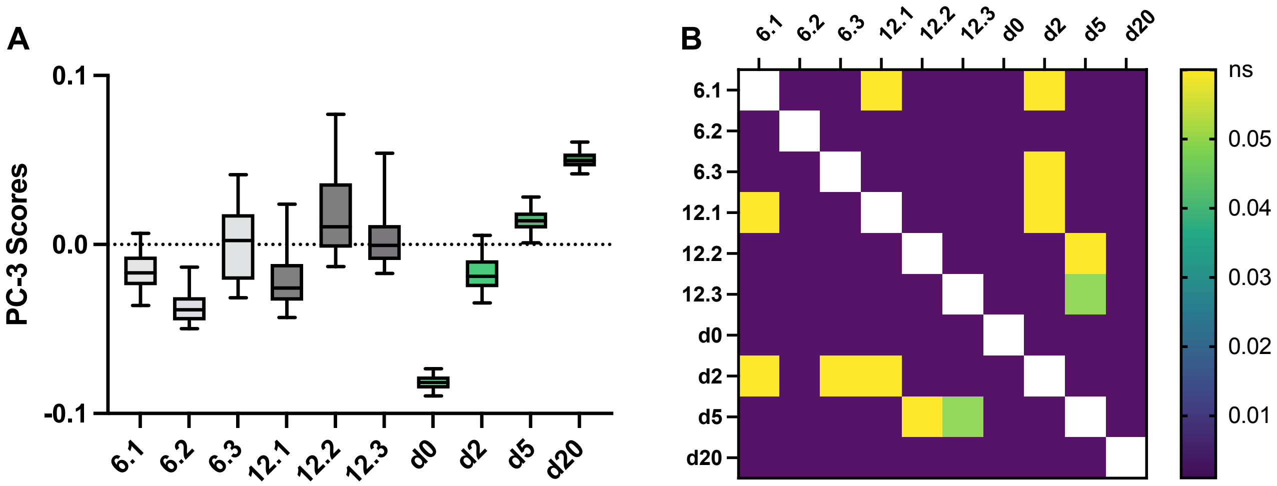

Supplementary figure S3. Multivariate analysis of PC-UPy in vivo degradation. (A) Comparison of average PC-3 score values per sample; mean ± SD **(B)** Heatmap of p values of multiple comparison via one-way ANOVA (of the data from A) demonstrates an overlap of the 6 months in vivo data with the 2-day in vitro degradation and the 2-5 days in vitro degradation with the 12 months explants.

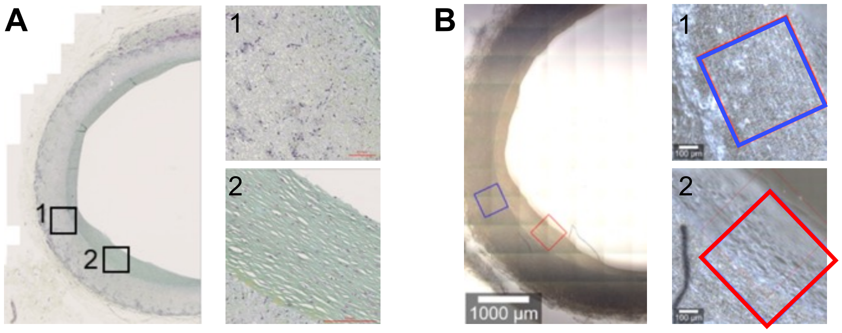

Supplementary figure S4. One of the 12-month explants just showed a thin adjacent intima layer. Histological staining (Movat Pentachrome) were provided by CVPath.

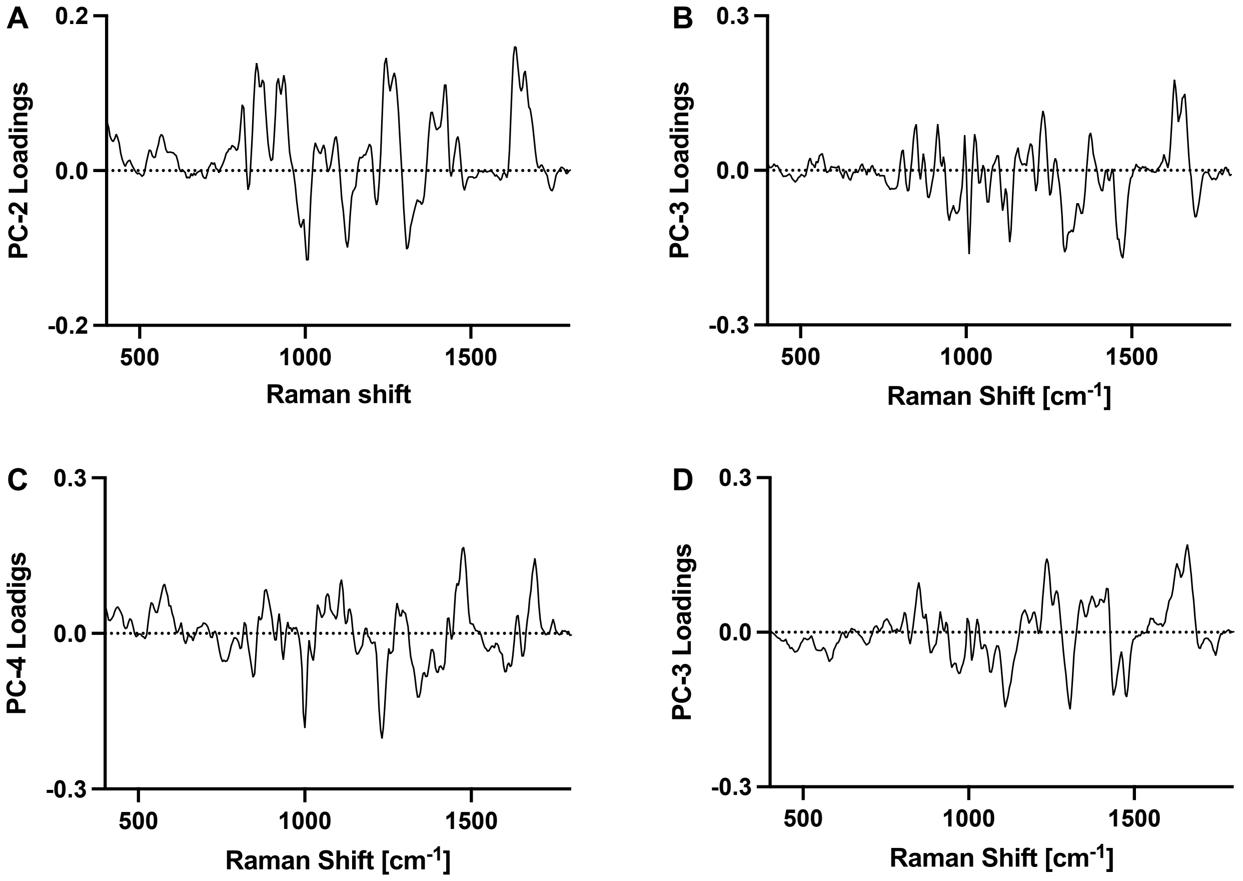

Supplementary figure S5. Multivariate analysis of collagen remodeling. Loadings plots corresponding to PCAs of **(A)** collagens of 6 vs 12-month implant region (ROI1), **(B)** collagens of 6 vs 12 month luminal region, **(C)** comparison between scaffold and luminal region in 6 month samples and **(D)** comparison between scaffold, interface and luminal region in 12 month samples.

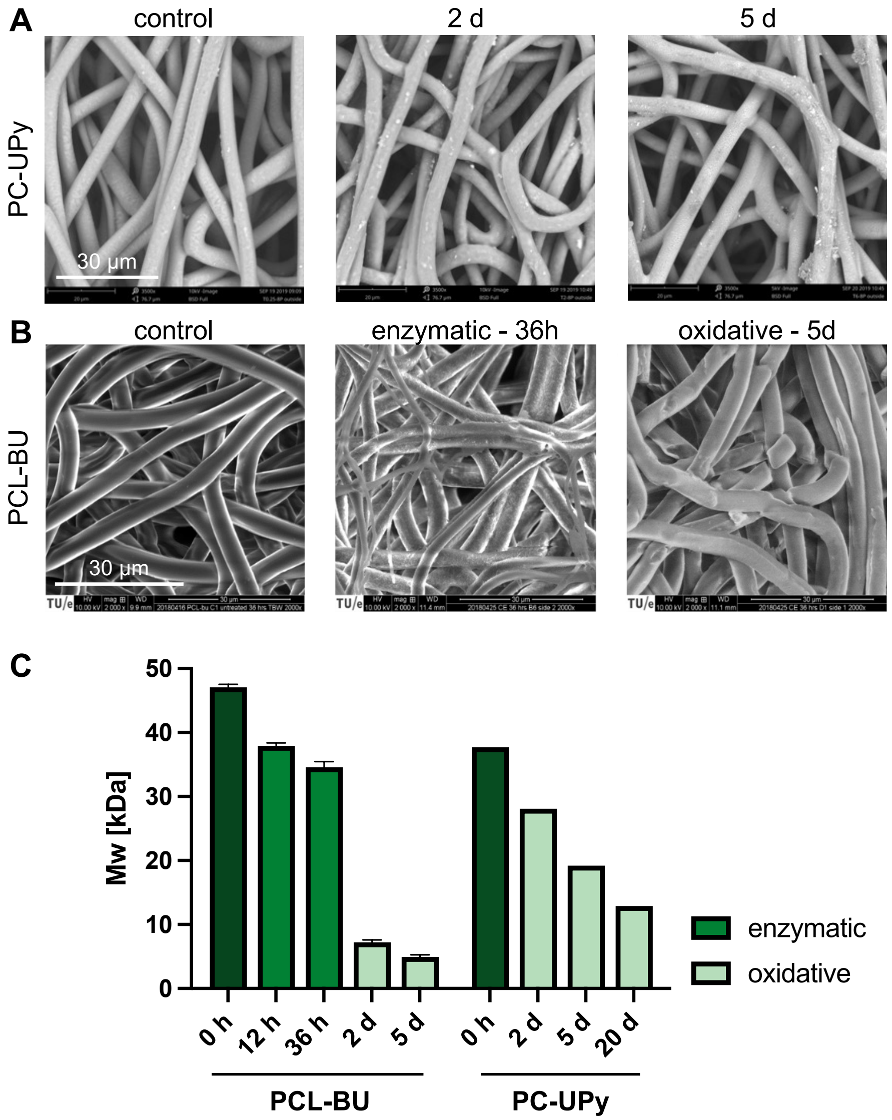

Supplementary figure S6. In vitro degradation determined by fiber morphology in SEM images of control, 2d and 5d oxidatively degraded PC-UPy scaffolds **(A),** control, 36h enzymatic or 5d oxidative degradation in PCL-BU scaffolds **(B)** and by analysis of mean molecular weight (Mw) by GPC **(C)**.
